# Spontaneous Resolution of a Bilateral Barrow Type D Indirect Carotid–Cavernous Fistula: A Rare Case Report and Literature Review

**DOI:** 10.3390/diagnostics16111594

**Published:** 2026-05-23

**Authors:** Madalina Totir, Ana Maria Dascalu, Ece Ergin, Bogdan Dorobat, Daniela Stana

**Affiliations:** 1 Faculty of Medicine, “Carol Davila” University of Medicine and Pharmacy Bucharest, 020021 Bucharest, Romania; madalina.totir@umfcd.ro; 2 Ophthalmology Department, Emergency University Hospital Bucharest, 050098 Bucharest, Romania; ece.ergin@drd.umfcd.ro (E.E.); dr.danielastana@yahoo.com (D.S.); 3 Angiography and Endovascular Therapy Department, Emergency University Hospital Bucharest, 050098 Bucharest, Romania; bdorobat@gmail.com

**Keywords:** carotid–cavernous fistula (CCF), indirect CCF, bilateral CCF, spontaneous closure, neuro-ophthalmology

## Abstract

**Background and Clinical Significance**: Bilateral carotid-cavernous fistulas are rare clinical entities characterized by heterogeneous clinical presentations and variable outcomes. **Case presentation**: We report the case of a 69-year-old woman with a three-month history of progressive bilateral conjunctival hyperemia, proptosis, intermittent diplopia, and a left eye abduction deficit. Her systemic history included long-standing arterial hypertension and previous thyroidectomy with stable substitutive therapy. Comprehensive ophthalmologic, neurologic, and endocrine evaluations excluded more common causes of orbital congestion, including thyroid eye disease, orbital cellulitis, cavernous sinus thrombosis, and idiopathic orbital inflammation. The patient denied any history of recent trauma. Digital subtraction angiography (DSA) confirmed a bilateral, low-flow, indirect Barrow type D carotid–cavernous fistula (CCF) supplied by dural branches of both the internal and external carotid arteries, with marked reflux into dilated superior ophthalmic veins. DSA was essential, as prior CT and MRI studies did not identify any vascular abnormalities. The patient was scheduled for transvenous embolization; however, during the follow-up she noted gradual improvement in her condition. Repeat pre-procedural angiography performed approximately two months later demonstrated complete spontaneous closure of all shunts, accompanied by full clinical resolution. **Conclusions**: Owing to the exceptional rarity of bilateral indirect CCFs and the added occurrence of spontaneous closure, this case expands the limited existing literature and emphasizes the diagnostic challenges and the need for individualized treatment timing supported by multidisciplinary evaluation in low-flow dural carotid–cavernous fistulas.

## 1. Introduction

Carotid–cavernous fistulas (CCFs) are abnormal arteriovenous communications between the carotid arterial system and the cavernous sinus [1,2,3]. Although carotid–cavernous fistulas are considered rare vascular lesions, their exact prevalence remains uncertain because of heterogeneous etiologies and limited population-based studies. Recent epidemiological data further support the uncommon incidence of CCFs in the general population, estimated at approximately 0.37 per 100,000 persons annually [3,4,5]. Bilateral carotid–cavernous fistulas are exceedingly uncommon, with only a limited number of cases reported in the literature, highlighting their rarity even among CCF presentations. In traumatic settings, a higher incidence has been reported in association with basilar skull fractures, particularly involving the middle cranial fossa [6]. This rare but challenging condition can lead to a variety of ocular and neurological symptoms, including red eye, diplopia, exophthalmos, and ophthalmoplegia [3,7]. The Barrow classification distinguishes direct high-flow fistulas (type A) from indirect low-flow dural fistulas (types B–D) based on arterial supply [7,8,9]. Type A fistulas form a direct communication between the internal carotid artery (ICA) and the cavernous sinus, most commonly following trauma [3,10]. Indirect types B–D are supplied by meningeal branches of the ICA, the external carotid artery (ECA), or both, and typically occur spontaneously in older individuals with vascular risk factors such as hypertension and atherosclerosis [3,4,9,10,11]. Most CCFs are unilateral; bilateral involvement is exceptionally uncommon, occurring in approximately 1–2% of all cases [12]. Spontaneous closure of indirect CCFs has been reported but remains infrequent and is particularly rare in bilateral disease [9,13,14]. Most previously reported bilateral Barrow type D CCFs required active intervention, whereas cases demonstrating complete spontaneous angiographic and clinical resolution remain exceptionally rare.

We report a rare case of a bilateral, low-flow, non-traumatic, indirect (Barrow type D) CCF that underwent complete spontaneous angiographic and clinical resolution prior to planned endovascular treatment. This case adds to the very limited literature on spontaneously resolving bilateral indirect CCFs and highlights the importance of individualized management and multidisciplinary evaluation.

## 2. Case Presentation

A 69-year-old woman first presented in February 2025 to a neurology specialist with intermittent headache and tinnitus. Her medical history included a total thyroidectomy in 1992 for benign nodules, grade III hypertension, dyslipidemia, and obesity. She denied any history of head trauma.

In March 2025, she was evaluated by an ophthalmologist after developing conjunctival hyperemia and a progressive onset of bilateral exophthalmos, more pronounced in the right eye. Neither the neurologist nor the ophthalmologist identified a clear etiology, and in April 2025 she was referred for cardiology and endocrinology assessment.

Endocrinological evaluation revealed a normal thyroid profile, including Thyroid-Stimulating Hormone (TSH) and free T4 levels, with negative thyroid autoantibodies (anti-TPO and TRAb). Cardiology evaluation confirmed previously known grade III essential hypertension with very high additional cardiovascular risk, dyslipidemia, and obesity. Carotid Doppler ultrasonography revealed no significant abnormalities.

In early May 2025, neurological re-evaluation documented a left sixth cranial nerve palsy with corresponding abduction deficit. Brain Magnetic Resonance Imaging (MRI) demonstrated a small meningioma of the right greater wing of the sphenoid, punctate demyelinating lesions consistent with a vascular–degenerative microangiopathic process, and normal findings regarding the orbits, internal and external carotid arteries, optic nerves, tracts, and chiasm. No abnormalities of the major arteriovenous structures were detectable on time-of-flight (TOF) sequences.

In late May 2025, the patient presented to our ophthalmology emergency unit with worsening ocular pain and diplopia. She was admitted to the ophthalmology service for further evaluation.

**Figure 1:**
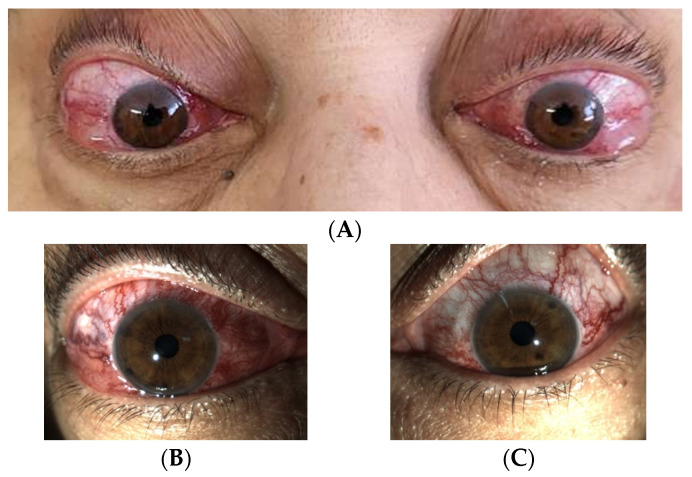
Ocular findings at initial presentation. (**A**). External photograph showing bilateral proptosis, orbital fat prolapse, and venous congestion. (**B**,**C**) Slit-lamp photographs of the right and left eyes illustrating diffuse chemosis and dilated episcleral vessels.

On examination, best-corrected visual acuity was 0.9 in right eye (RE) and 1.0 left eye (LE). Intraocular pressure was elevated bilaterally (25 mmHg RE, 26 mmHg LE); therefore, topical antiglaucoma treatment with a fixed brinzolamide–timolol combination (Azarga^®^_,_ Novartis Manufacturing NV/Alcon-Couvreur N.V., Puurs, Belgium) was initiated twice daily. Ocular motility was full in the RE, while the LE demonstrated marked abduction deficit. A thorough anterior segment evaluation revealed bilateral episcleral venous congestion, diffuse conjunctival chemosis, and marked bilateral proptosis with orbital fat prolapse (Figure 1A–C).

**Figure 2:**
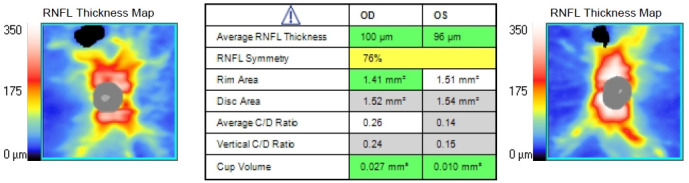
Optical coherence tomography of the optic nerve showing normal retinal nerve fiber layer thickness without evidence of optic neuropathy.

Comprehensive posterior segment evaluation, including fundus examination and optic nerve optical coherence tomography (OCT) (Figure 2), excluded optic neuropathy or structural compromise attributable to orbital venous congestion.

**Figure 3:**
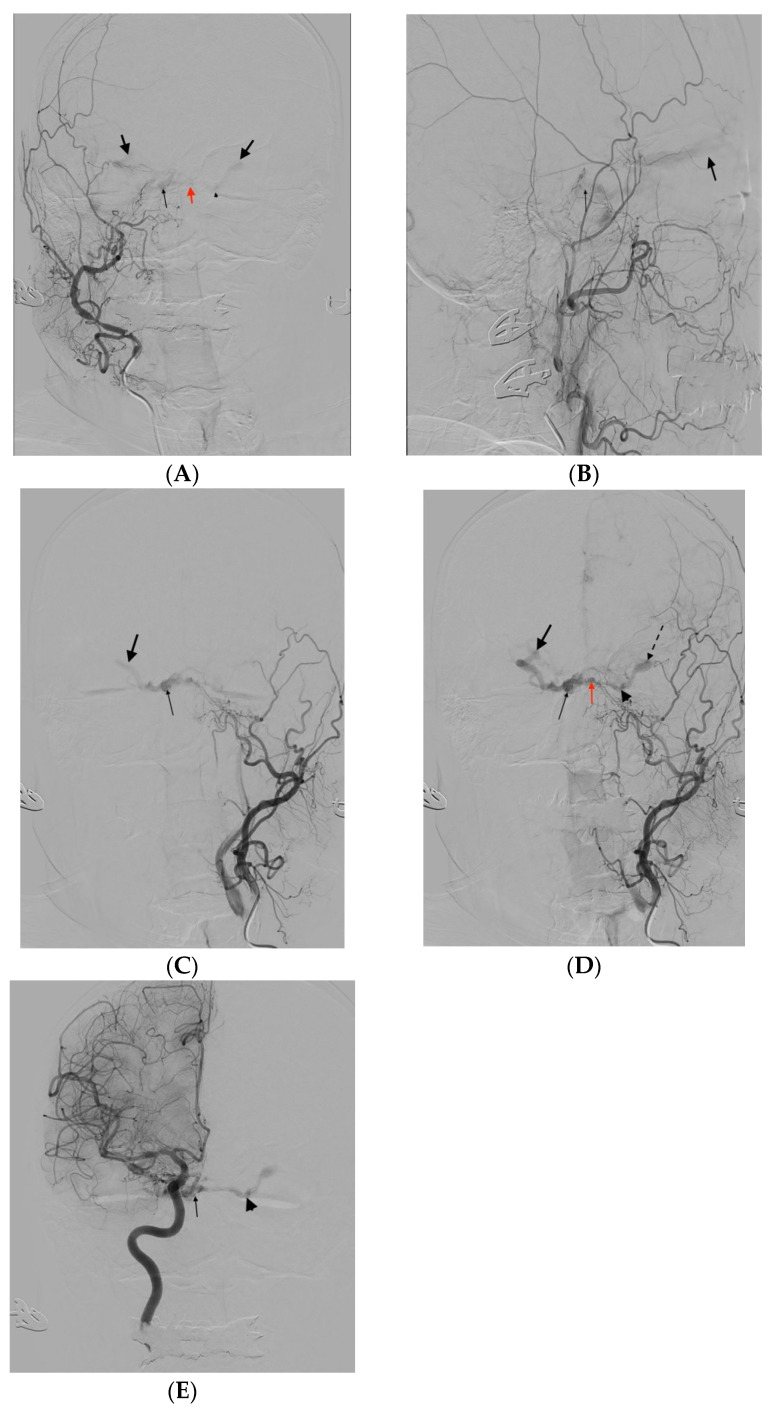

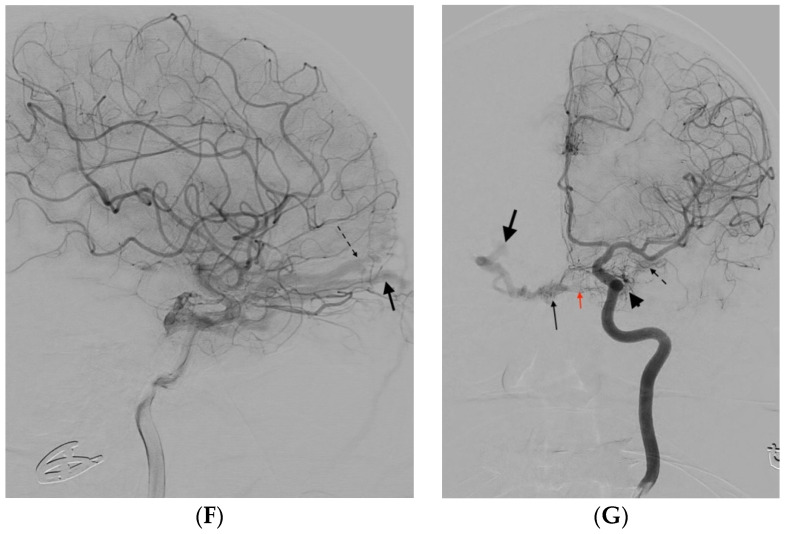
Digital subtraction angiography demonstrating bilateral indirect Barrow type D carotid–cavernous fistulas. (**A**,**B**) Right external carotid artery (ECA) injection in frontal (**A**) and lateral (**B**) views, showing dural arterial feeders to the cavernous sinus with early opacification of the right cavernous sinus (black arrow), opacification of the intercavernous sinus (red arrow) with subsequent left cavernous sinus filling (arrowhead), and retrograde drainage of the ipsilateral ophthalmic vein from each cavernous sinus (solid arrow). (**C**,**D**) Left external carotid artery (ECA) injection demonstrating similar dural supply to the right cavernous sinus (black arrow) with venous drainage into a dilated right superior ophthalmic vein (solid arrow). Opacification of the right cavernous sinus results in secondary filling of the left cavernous sinus (arrowhead) via the intercavernous sinus (red arrow), with associated drainage into a dilated left superior ophthalmic vein (dashed arrow). (**E**) Right internal carotid artery (ICA) injection revealing meningeal branches contributing to the fistula, early filling of the right cavernous sinus (black arrow) and left cavernous sinus via intercavernous connection (arrowhead), confirming the indirect nature of the shunt. (**F**,**G**) Left internal carotid artery (ICA) injection in frontal (**G**) and lateral (**F**) views, showing corresponding dural feeders filling the right cavernous sinus (black arrow) with ophthalmic reflux (solid arrow) and intercavernous connection (red arrow) filling the left cavernous sinus (arrowhead), and left drainage of the ophthalmic vein (dashed arrow) completing the angiographic characterization of bilateral Barrow type D fistulas.

Given the clinical features suggestive of orbital venous engorgement and normal prior endocrine and neurologic evaluations, including laboratory testing, digital subtraction angiography (DSA) was performed. DSA demonstrated bilateral, low-flow, indirect Barrow type D CCFs supplied by dural branches of both the ICA and ECA, with prominent reflux into dilated superior ophthalmic veins (Figure 3A–G).

Endovascular transvenous embolization was scheduled approximately six weeks later, consistent with standard management strategies for low-flow dural fistulas, after a reasonable disclosure regarding potential risks and benefits [7,9,15].

Considering hypercoagulability as a potential contributing factor, the patient underwent thrombophilia evaluation, including protein C, protein S, antithrombin III levels, Factor V Leiden mutation, prothrombin gene mutation analysis, and antiphospholipid antibody testing (lupus anticoagulant, anticardiolipin, and anti-β2 glycoprotein I antibodies). However, no evidence of an underlying hypercoagulable state was identified.

Satisfactory intraocular pressure control was subsequently achieved with conventional topical therapy during the initial evaluation period while definitive fistula management was being planned.

**Figure 4:**
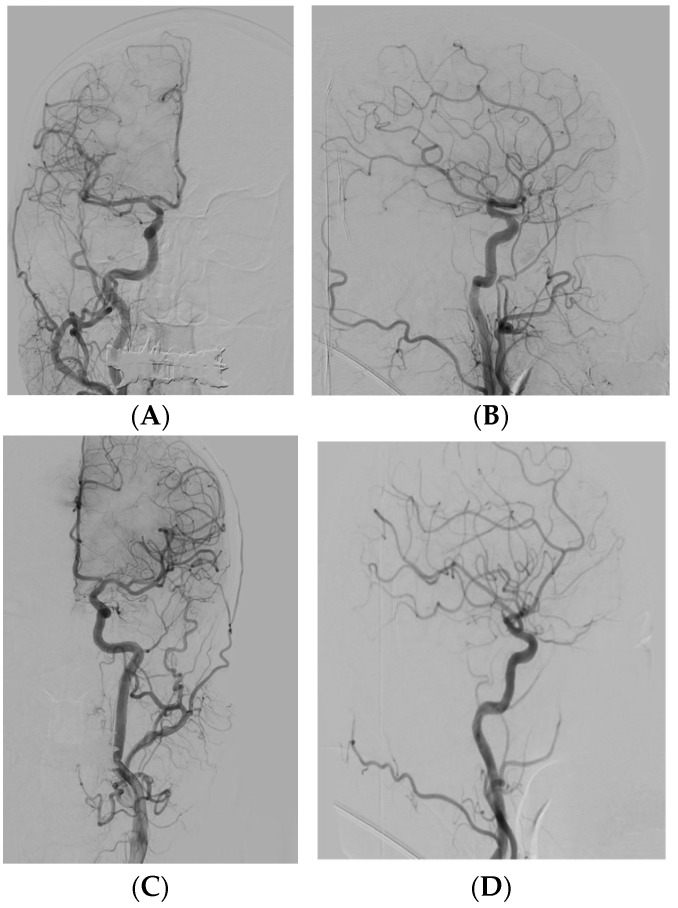
Follow-up digital subtraction angiography demonstrating complete spontaneous closure of bilateral indirect carotid–cavernous fistulas. (**A**,**B**) Right common carotid artery (CCA) injection showing no residual arteriovenous shunting. There is no early opacification of the cavernous sinus or superior ophthalmic vein, confirming complete spontaneous occlusion on the right side. (**C**,**D**) Left common carotid artery (CCA) injection demonstrating the same findings, with normal arterial and venous filling patterns and absence of any persistent fistulous communication or venous reflux.

However, repeat pre-procedural angiography performed approximately two months after the initial DSA demonstrated complete spontaneous closure of all arteriovenous communications, with no residual shunting or venous reflux (Figure 4A–D).

**Figure 5:**
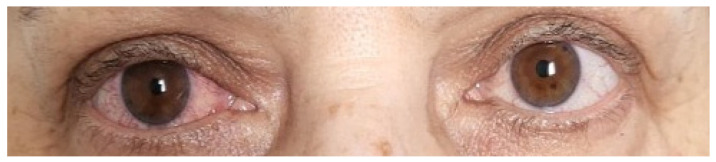
Clinical appearance following spontaneous closure of bilateral indirect carotid–cavernous fistulas.

By this time, the patient exhibited complete clinical resolution, including normalization of intraocular pressure and resolution of exophthalmos, conjunctival congestion, and diplopia, with only mild residual conjunctival hyperemia in the right eye (Figure 5).

**Figure 6:**
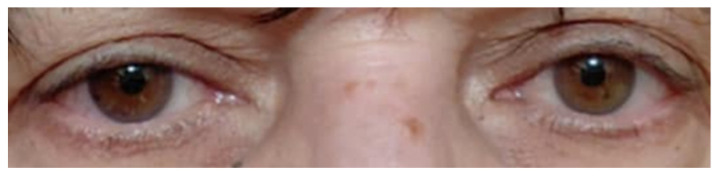
Clinical appearance at 4-month follow-up after spontaneous closure of bilateral indirect carotid–cavernous fistulas.

No recurrence of symptoms was noted on subsequent follow-up. Clinical examination performed in December 2025 confirmed sustained resolution, with stable ocular findings and no evidence of disease recurrence (Figure 6).

## 3. Discussions

The pathophysiology of CCFs is characterized by the abnormal arterial flow into the cavernous sinus, which houses cranial nerves III, IV, V1, V2, and VI, and receives venous drainage from the orbit via the superior and inferior ophthalmic veins [3]. High- or moderate-pressure shunting of arterial blood into this venous system causes orbital venous congestion, resulting in proptosis, conjunctival chemosis, and arterialized episcleral vessels. Elevated episcleral venous pressure impairs aqueous humor outflow, causing secondary open-angle glaucoma, while retinal venous congestion may lead to venous tortuosity or, rarely, central retinal vein occlusion [16,17]. Rho kinase inhibitors have recently emerged as a potential therapeutic option in ocular hypertension associated with elevated episcleral venous pressure [18]. However, in our patient, satisfactory intraocular pressure control was achieved with conventional topical therapy, and rho kinase inhibitors were not available in our country at the time of management. Compression of the cranial nerves within the cavernous sinus leads to diplopia, the VI nerve being most frequently involved. In carotid–cavernous fistulas, Hess screen testing may demonstrate patterns of restrictive extraocular motility secondary to cranial nerve dysfunction and orbital venous congestion. In the present case, a Hess screen test was not performed, as the diagnosis was established based on clinical ophthalmologic examination and confirmed by digital subtraction angiography findings. If left untreated, vision loss may occur due to secondary glaucoma, venous stasis, and, in severe or prolonged cases, ischemic optic neuropathy [13].

The differential diagnosis of indirect carotid–cavernous fistulas may be particularly challenging in bilateral presentations, where orbital congestion can mimic endocrine, inflammatory, infectious, venous, or infiltrative disorders [2,3]. Infectious and inflammatory conditions such as orbital cellulitis and idiopathic orbital inflammatory disease can present with painful proptosis and restricted ocular movements [3,19]. Thyroid eye disease may resemble CCFs but typically lacks orbital bruit, corkscrew episcleral vessels, and angiographic vascular abnormalities [3,20]. Neoplastic causes involving the cavernous sinus or orbital apex, such as meningioma or metastatic disease, may produce gradual cranial nerve deficits and proptosis [3]. Acute glaucomatous conditions associated with painful red eye and elevated intraocular pressure may clinically resemble CCF but do not cause pulsatile proptosis or vascular bruits [17]. Helpful clinical clues supporting CCF diagnosis include pulsatile proptosis, orbital bruit, arterialized corkscrew conjunctival vessels, chemosis, elevated intraocular pressure, diplopia associated with cranial nerve VI palsy, and tinnitus [2,3].

The Barrow classification provides a structured framework for distinguishing CCF subtypes according to arterial supply and flow characteristics (4–6). Direct type A fistulas form a high-flow connection between the cavernous ICA and cavernous sinus, frequently following trauma [3,10]. In contrast, indirect types B–D arise from dural branches of the ICA, ECA, or both and typically exhibit low-flow hemodynamics consistent with their spontaneous, non-traumatic nature [3,9]. Table 1.

Indirect CCFs predominantly affect postmenopausal women and are associated with vascular risk factors such as hypertension, atherosclerosis, diabetes, vascular dysfunction or collagen vascular diseases. In these cases, CCFs appear due to a dural artery rupture, after Valsalva maneuver or minor stress [3,4,10,11]. Epidemiological data indicate that indirect CCFs account for approximately 60–70% of all cases, whereas direct CCFs represent about 30–40% and are more commonly related to trauma [3,10,12]. The majority of CCFs are unilateral, with more than 80% of cases involving a single side, while bilateral involvement remains distinctly uncommon [10]. Although spontaneous closure has been reported in isolated indirect CCFs, it is rare and has been only exceptionally described in bilateral disease [9,13,14].

Although spontaneous closure has been reported in isolated indirect carotid–cavernous fistulas, it remains rare and has been only exceptionally described in bilateral disease. Most reported cases of bilateral Barrow type D CCFs have required active intervention, even in the absence of trauma [21,22,23,24]. Non-traumatic bilateral indirect CCFs treated with endovascular embolization have been described by several authors [16,21,22,23,24,25,26,27,28,29,30,31,32,33,34,35,36,37,38], while Khan et al. [29] reported a bilateral indirect CCF initially managed conservatively with manual carotid compression. In contrast, spontaneous angiographic closure has been reported primarily in traumatic bilateral cases, such as that described by Ke et al. [30,31]. However, persistent raised IOP and episcleral vein pressure led to permanent vision loss in the most affected eye. Truly spontaneous resolution of bilateral, non-traumatic, indirect Barrow type D CCFs without any intervention remains exceedingly rare, with only isolated cases reported in the literature, including the case described by Baig et al. [13] (Table 2). What distinguishes the present case is the combination of bilateral indirect Barrow type D angioarchitecture, absence of trauma, delayed diagnosis despite extensive prior investigations, and complete spontaneous angiographic and clinical resolution without any intervention.

Clinically, CCFs may produce a broad spectrum of ocular and neuro-ophthalmic manifestations, depending on flow dynamics and venous drainage, including conjunctival hyperemia, chemosis, proptosis, diplopia, elevated intraocular pressure, and sixth nerve palsy [2,30,31]. In severe cases, CCFs may be complicated by optic neuropathy or exposure keratopathy [2,30]. In the present case, although the patient exhibited significant orbital venous congestion and cranial nerve dysfunction, no vision-threatening complications developed. Differential diagnosis for patients presenting with such signs should take CCFs into account even if they are an uncommon diagnosis. CCFs pose significant diagnostic challenges, often leading to delayed diagnosis. In our case a prior history of thyroidectomy contributed to diagnostic difficulty, slowing recognition of bilateral indirect CCF.

Although most CCFs are unilateral, bilateral cases have been reported, particularly in indirect dural fistulas. Bilateral CCFs may present spontaneously in elderly women with hypertension or connective tissue disorders, following trauma, or rarely iatrogenically after vascular procedures. Clinically, bilateral involvement can result in more severe and symmetric orbital venous congestion, bilateral proptosis, chemosis, elevated intraocular pressure, and diplopia affecting both eyes. Bilateral CCFs carry a higher risk of visual compromise and cortical venous reflux, increasing the potential for intracranial complications such as hemorrhage or venous infarction.

Diagnosis requires a high index of suspicion in patients presenting with unilateral or bilateral red eyes, proptosis, diplopia, and ocular bruits. Neuroimaging with CT or MRI can demonstrate enlargement of the superior ophthalmic vein, cavernous sinus expansion, and extraocular muscle swelling.

**Figure 7:**
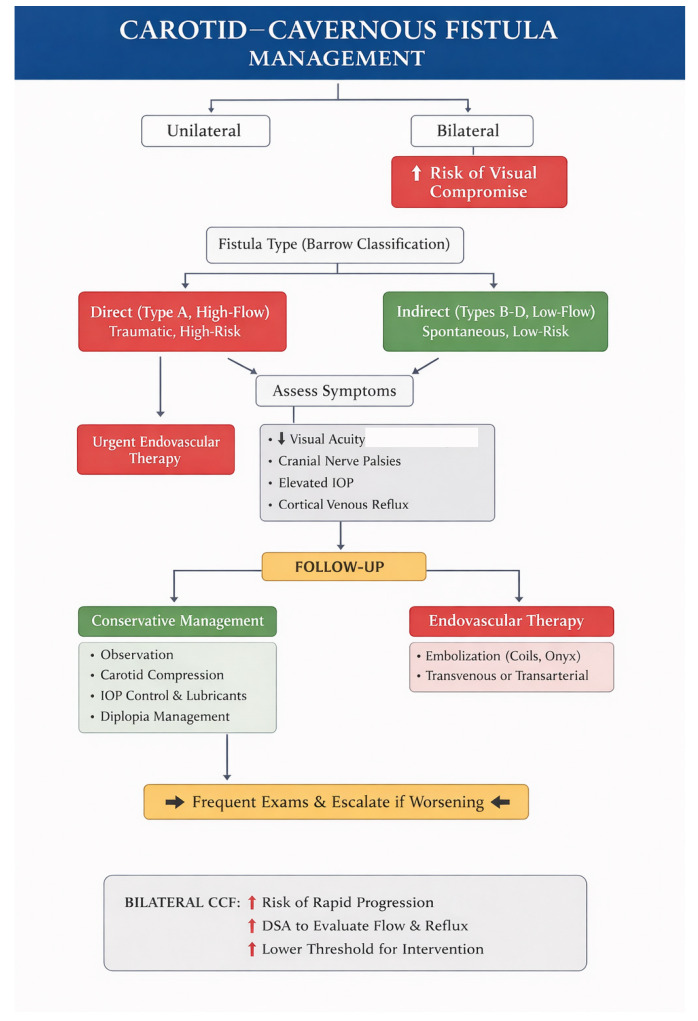
Carotid—cavernous fistula—a systematic approach. Footnote: ↑ increase; ↓ decrease.

Non-invasive imaging modalities may demonstrate limited sensitivity in low-flow indirect carotid–cavernous fistulas, particularly in bilateral lesions with subtle hemodynamic abnormalities [3,29]. Routine MRI, MRA, TOF sequences, Doppler ultrasonography, and even CT imaging may fail to identify small dural shunts or early venous drainage abnormalities, potentially contributing to delayed diagnosis [3,9,17,29]. In contrast, digital subtraction angiography remains the diagnostic gold standard because of its superior ability to characterize fistula angioarchitecture, arterial feeders, venous drainage pathways, and flow dynamics [3,11]. DSA in our patient confirmed bilateral indirect Barrow type D fistulas with reflux into dilated superior ophthalmic veins. Effective management of CCFs requires a systematic approach integrating clinical assessment, imaging techniques, and individualized endovascular therapy, as summarized in the proposed diagnostic and therapeutic algorithm presented in Figure 7 [32,33,34].

Endovascular embolization, particularly via a transvenous approach, is the recommended treatment for most symptomatic indirect CCFs, given its high efficacy, low morbidity, and ability to prevent long-term complications [11,14,21]. However, our patient demonstrated complete angiographic and clinical resolution before planned treatment—an outcome that has been reported only rarely and most often in traumatic or unilateral cases, with spontaneous closure in non-traumatic bilateral disease remaining exceptionally uncommon [13,31]. Given the exceptional rarity of bilateral carotid–cavernous fistulas, the existing literature is largely limited to isolated case reports and small case series, restricting comparative analysis of clinical presentation and angiographic pattern [32,35]. This case illustrates the diagnostic challenges and the potential for complete spontaneous resolution in bilateral indirect carotid–cavernous fistulas and underscores the importance of careful clinical surveillance and individualized therapeutic decision-making in selected low-flow dural lesions.

The exact mechanism underlying spontaneous closure in the present case cannot be determined with certainty. Proposed mechanisms described in previous reports include spontaneous thrombosis secondary to low-flow hemodynamics and venous stasis, progressive reduction in the pressure gradient across the fistulous communication, and endothelial injury or arterial spasm induced during angiographic catheterization procedures [11,13,30]. In indirect low-flow lesions, these mechanisms may contribute to progressive reduction and eventual cessation of arteriovenous shunting [11,13].

Hypercoagulable states have also been proposed as potential contributing factors in spontaneous indirect carotid–cavernous fistula formation and thrombosis [3,9,36,37]. Laboratory investigations in indirect CCFs may assist in identifying predisposing systemic or vascular conditions rather than confirming the fistula itself [3,36]. In selected cases, particularly when connective tissue or vascular disorders such as Ehlers–Danlos syndrome or vasculitis are suspected, additional investigations including genetic testing or autoimmune markers (ANA/ANCA) may be considered [3,36]. Evaluation for thrombophilia may also be appropriate in patients with suspected hypercoagulable states or venous thrombosis [36]. In our patient, however, no abnormal condition associated with hypercoagulation and thrombosis was identified.

Conservative management may be considered for low-flow, indirect CCFs with mild symptoms, stable vision, and no optic neuropathy or cortical venous reflux. Many such fistulas may spontaneously thrombose due to low shunt pressure and altered venous hemodynamics [11,13]. A low gradient between ICA and the fistula may favor blood flow stagnation and thrombosis. Conservative management with manual carotid compression has been described in selected low-flow indirect carotid–cavernous fistulas, with some reports demonstrating closure in up to 30% of unilateral indirect lesions following intermittent carotid–jugular compression at the level of the carotid bulb [3,32,38,39]. However, its efficacy remains variable and careful patient selection is required, particularly in bilateral cases associated with ocular hypertension or cranial nerve dysfunction [3,32]. Observation may be considered in selected patients with stable visual function, tolerable symptoms, absence of progressive cranial nerve palsies, and no evidence of optic neuropathy [3,32]. In the present case, manual carotid compression was not attempted because of persistent symptoms, elevated intraocular pressure, bilateral venous congestion, and cranial nerve involvement, while close ophthalmologic monitoring was maintained throughout follow-up. Furthermore, bilateral indirect fistulas often demonstrate complex dural arterial feeders and impaired venous drainage, potentially reducing the efficacy of intermittent carotid compression and increasing the theoretical risk of cerebral hypoperfusion, and ischemic events, especially in older patients who commonly have atherosclerotic carotid disease [31,38,39]. The other presumed mechanism is that navigation of microguide wires and catheters through small-sized fistulas during angiography could induce closure, due to arterial spasm, endothelial damage, or arterial dissection [13].

Conservative care requires close monitoring of visual acuity, color vision, visual fields, ocular motility, and intraocular pressure. Symptomatic measures include ocular surface lubrication, intraocular-pressure-lowering drops, and temporary management of diplopia with patching or prisms [5,34,40]. Immediate endovascular therapy is indicated if vision declines, optic neuropathy develops, intraocular pressure rises uncontrollably, or cranial nerve deficits progress. In bilateral cases, conservative management may be employed only with close follow-up due to the higher risk of rapid deterioration [40].

Prognosis in carotid–cavernous fistulas depend largely on fistula type, venous drainage pattern, duration of symptoms before diagnosis, presence of optic neuropathy, and response to treatment or spontaneous closure [3,30,34,41]. Indirect low-flow fistulas generally demonstrate a more favorable prognosis, particularly in patients with preserved visual acuity and absence of cortical venous reflux [3,34]. Ocular complications such as retinal ischemia, secondary glaucoma, optic neuropathy, progressive ophthalmoplegia, severe proptosis with exposure keratopathy, and persistent cranial nerve dysfunction may lead to significant visual morbidity if diagnosis or treatment is delayed [3,17,34,41]. In traumatic CCFs, visual loss may additionally occur secondary to associated ocular or optic nerve injury [31,41]. Although CCFs are usually not life-threatening, serious complications such as cerebral infarction, intracranial hemorrhage, or subarachnoid hemorrhage secondary to fistula rupture may rarely occur [3,34,41]. In the present case, preserved visual acuity, absence of optic neuropathy, and complete spontaneous angiographic closure were associated with a favorable long-term clinical outcome.

The main strength of this manuscript lies in its report of an exceptionally rare clinical entity—bilateral, non-traumatic, low-flow Barrow type D carotid–cavernous fistulas—with the even more uncommon outcome of complete spontaneous angiographic and clinical resolution prior to intervention. To our knowledge, such a combination of bilateral involvement and full spontaneous closure remains exceedingly scarce in the existing literature. In addition, this case underscores critical diagnostic and clinical decision-making challenges in low-flow dural fistulas, particularly the limitations of non-invasive imaging in early detection and the importance of DSA in establishing the diagnosis. The report further contributes by synthesizing current evidence on natural history variability, reinforcing that selected indirect fistulas may demonstrate unpredictable spontaneous resolution despite conventional indications for endovascular therapy.

## 4. Conclusions

Bilateral indirect carotid–cavernous fistulas are exceptionally rare and may present with nonspecific orbital symptoms, often delaying diagnosis. This case highlights the key clinical value of maintaining a high index of suspicion in patients with persistent orbital congestion and inconclusive non-invasive imaging, with digital subtraction angiography remaining essential for definitive diagnosis. Importantly, it demonstrates that low-flow Barrow type D fistulas may undergo complete spontaneous resolution even in bilateral disease, underscoring the need for individualized, multidisciplinary management and careful timing of intervention.

## Figures and Tables

**Table 1:** Detailed Barrow classification for carotid–cavernous fistulas (adapted from Barrow et al., 1985 [7], and Henderson & Miller, 2018 [3]).

Barrow Type	Flow Characteristics	Arterial Supply	Typical Etiology	Key Features
A	High-flow, direct	ICA → cavernous sinus	Traumatic	Acute onset, severe proptosis, chemosis, bruit
B	Low-flow, indirect	Meningeal branches of ICA	Spontaneous	Gradual onset, milder symptoms
C	Low-flow, indirect	Meningeal branches of ECA	Spontaneous	Similar to type B; ECA feeders
D	Low-flow, indirect	Meningeal branches of ICA + ECA	Spontaneous	May be bilateral; rare spontaneous closure

**Table 2:** Reported cases of bilateral carotid–cavernous fistulas (CCFs)—clinical management and outcomes.

Author, Year	Type (Barrow)	Etiology	Clinical Signs	Management	Outcome
Churojana et al., 2001 [31]	Direct (high-flow)	Traumatic	Bilateral proptosis, chemosis, subconjunctival hemorrhage	Conservative observation	Complete spontaneous angiographic closure
Ke et al., 2017 [30]	Not specified (likely indirect)	Traumatic	Bilateral red eyes, mild proptosis, elevated IOP	Conservative observation, IOP control	Gradual improvement; right eye NLP at 2 months
Docherty et al., 2018 [27]	Not specified	Traumatic	Bilateral proptosis, chemosis, diplopia, ocular bruit	Endovascular embolization	Resolution of proptosis and diplopia; vision preserved
Liang et al., 2021 [28]	Direct	Traumatic	Bilateral proptosis, ocular bruit, diplopia	Endovascular embolization	Complete resolution; vision preserved
Gasparian & Chalam, 2021 [21]	Indirect	Spontaneous	Bilateral proptosis, chemosis, diplopia, mild IOP elevation	Endovascular coil embolization	Resolution of ocular signs; CN recovery over weeks
Baig et al., 2021 [13]	Indirect (type D)	Spontaneous	Bilateral proptosis, chemosis, diplopia, blurred vision	No intervention	Complete spontaneous resolution
Pellegrini et al., 2022 [22]	Direct	Spontaneous	Bilateral proptosis, chemosis, diplopia, CN VI palsy	Endovascular embolization	Rapid improvement; gradual CN recovery
Shah et al., 2022 [16]	Indirect (type D)	Spontaneous	Bilateral red eyes, chemosis, diplopia	Endovascular embolization	Improvement in ocular signs; partial CN recovery
Sharma et al., 2022 [23]	Indirect	Spontaneous	Bilateral proptosis, chemosis, diplopia, elevated IOP	Endovascular embolization	Symptom resolution; no permanent visual loss
Camara et al., 2024 [25]	Direct	Traumatic	Bilateral proptosis, conjunctival congestion, diplopia	Endovascular embolization	Resolution of proptosis and IOP; full visual recovery
Balodis et al., 2024 [24]	Indirect (type D, low-flow)	Spontaneous	Bilateral chemosis, episcleral injection, elevated IOP	Endovascular embolization guided by 3D TOF-MRA	Clinical and angiographic resolution

Footnote: CCF, carotid–cavernous fistula; CN, cranial nerve; IOP, intraocular pressure; NLP, no light perception; TOF-MRA, time-of-flight magnetic resonance angiography.

## Data Availability

The original contributions presented in this study are included in the article. Further inquiries can be directed to the corresponding author.
